# Inequities in breast cancer treatment in sub-Saharan Africa: findings from a prospective multi-country observational study

**DOI:** 10.1186/s13058-019-1174-4

**Published:** 2019-08-13

**Authors:** Milena Foerster, Benjamin O. Anderson, Fiona McKenzie, Moses Galukande, Angelica Anele, Charles Adisa, Annelle Zietsman, Joachim Schuz, Isabel dos Santos Silva, Valerie McCormack

**Affiliations:** 1 Section of Environment and Radiation, International Agency for Research on Cancer (IARC), 150 Cours Albert Thomas, Lyon, France; 2 Fred Hutchinson Cancer Research Center, Seattle, WA USA; 3 Makerere University, Kampala, Uganda; 4 Federal Medical Centre Owerri, Owerri, Nigeria; 5 Abia State University Teaching Hospital, Aba, Nigeria; 6 Windhoek Central Hospital, Windhoek, Namibia; 7 Department of Non-Communicable Disease Epidemiology, London School of Hygiene & Tropical Medicine, London, UK

**Keywords:** Africa, Breast cancer treatment, Surgery, Cancer, Radiotherapy, Chemotherapy

## Abstract

**Background:**

Improving breast cancer survival in sub-Saharan Africa (SSA) is urgently needed, requiring early diagnosis and improved access to treatment. However, data on the types of and barriers to receiving breast cancer therapy in this region are limited and have not been compared between different SSA countries and treatment settings.

**Methods:**

In different health care settings across Uganda, Nigeria and Namibian sites of the prospective African Breast Cancer - Disparities in Outcomes cohort study, we assessed the percentage of newly diagnosed breast cancer patients who received treatment (systemic, surgery and/or radiotherapy) for cancer and their socio-demographic and clinical determinants. Treatment data were systematically extracted from medical records, as well as self-reported by women during 6-month follow-up interviews, and were used to generate a binary indicator of treatment received within 12 months of diagnosis (yes/no), which was analysed via logistic regression.

**Results:**

Of 1325 women, cancer treatment had not been initiated treatment within 1 year of diagnosis for 227 (17%) women and 185 (14%) of women with stage I–III disease. Untreated percentages were highest in two Nigerian regional hospitals where 38% of 314 women were not treated (32% among stage I–III). At a national referral hospital in Uganda, 18% of 430 women were not treated (15% among stage I–III). In contrast, at a cancer care centre in Windhoek, Namibia, where treatment is provided free to the patient, all non-black (100%) and almost all (98.7%) black women had initiated treatment. Percentages of untreated women were higher in women from lower socio-economic groups, women who believed in traditional medicine and, in Uganda, in HIV+ women. Self-reported treatment barriers confirmed treatment costs and treatment refusal as contributors to not receiving treatment.

**Conclusions:**

Financial support to ensure treatment access and education of treatment benefits are needed to improve treatment access for breast cancer patients across sub-Saharan Africa, especially at regional treatment centres, for lower socio-economic groups, and for the HIV-positive woman with breast cancer.

**Electronic supplementary material:**

The online version of this article (10.1186/s13058-019-1174-4) contains supplementary material, which is available to authorized users.

## Background

In sub-Saharan Africa (SSA), breast cancer was the most common cancer diagnosed in women in 2018 [1]. A key breast cancer control priority in this region is to improve survival rates which are now among world’s lowest [2]. To do this, as per the Breast Health Global Initiative’s recommended phased implementation approach, SSA countries need to develop and strengthen programs to ensure the early diagnosis of symptomatic breast cancer, in parallel with improvements in timely access to appropriate treatment. Whilst there are several studies on the predominantly advanced stage at breast cancer diagnosis in SSA [3], there are limited data on breast cancer treatment gaps from this region.

Standardised breast cancer treatment guidelines tailor therapy to key tumour features: stage at diagnosis, and expression of oestrogen, progesterone and HER2 receptors, for which curative treatment involves some combination of surgery, radiotherapy and systemic (drug) therapies. Recognising that the majority of countries lack the capacity to deliver state-of-the-art therapeutics, the National Comprehensive Cancer Network (NCCN) has provided resource-stratified oncology care guidelines, including a SSA-specific “harmonised” version [4]. Indeed, barriers to accessing oncology care are seemingly ubiquitous in parts of SSA. At the health system level, oncology treatment is lacking and waiting times for chemotherapy and, if available, radiotherapy are long [5]. Several further setting-specific factors may influence treatment acceptability and access. At the patient level, fear of disfigurement, of stigmatisation or of treatment side-effects have been documented [6, 7], whilst the risks of incurring catastrophic health expenditures are large [8, 9]. The influence of traditional and spiritual healers on receipt of conventional cancer care has not been quantified, and finally, in HIV-endemic regions of SSA, the evidence base on management of the HIV-positive woman with breast cancer is very limited [10].

In 2014, the International Agency for Research on Cancer (IARC) and the London School of Hygiene and Tropical Medicine (LSHTM) launched the African Breast Cancer - Disparities in Outcomes (ABC-DO) study, a cohort of over 2000 women newly diagnosed with breast cancer in five SSA countries. This ongoing prospective observational study collected multidimensional data throughout the diagnostic, treatment and survivorship period [11]. In analyses to date, advanced stage at diagnosis was associated with delays to diagnosis, lower socio-economic status and low levels of breast cancer awareness [12, 13]. Continuing along this breast cancer journey, we herein examine cancer treatment access and its determinants. The value of this analysis is to identify SSA-wide and setting-specific opportunities for systematic improvements of breast cancer care which, when coupled with early diagnosis, will translate into averted breast cancer deaths.

## Methods

### Study design and setting

ABC-DO is a prospective multi-centric hospital-based study of survival after breast cancer diagnosis in five SSA countries. A detailed protocol has been published [11]. During September 2014 to early 2016, all women aged 18 and older presenting in the participating hospitals and suspected of having breast cancer (either clinically or through histological confirmation) were invited to participate. Benign cases were excluded from participation after diagnostic workup. Across all sites, 99% of invited women (*n* = 2265) agreed to participate. In the present analysis, after excluding 7 women with no treatment data, the partaking hospitals included 1335 women from (i) Windhoek Central Hospital’s AB May Cancer Care Centre in Namibia (*n* = 502, among these *n* = 398 black women and *n* = 104 non-black women); (ii) Uganda Cancer Institute and Mulago Hospital, Kampala (*n* = 430) and (iii) the Federal Medical Centre, Owerri, and Abia State University Teaching Hospital, Aba (*n* = 313, combined as Nigeria public), and, the only private setting, the Maranatha clinic, Aba (*n* = 80), Nigeria. Two ABC-DO countries that lacked complete or harmonised treatment information at the time of analysis were not included, i.e. Zambia (*n* = 207) owing to a later recruitment period and South Africa (*n* = 716) due to a different data capture system. For the three included countries, the catchment populations, and breast cancer treatment options and their approximate costs are listed in Table 1. The sites include diverse public-sector cancer care providers, notably two regional hospitals in Nigeria, a national referral general hospital in Uganda and a specialised national cancer care centre in Namibia, with varying out-of-pocket care costs to the patient. Due to these differences, treatment access is thought to reflect each site as an example of the cancer treatment setting, rather than as representative of the country as a whole.

**Table 1:** Study hospitals, their catchment populations, available treatments and respective approximate patient out-of-pocket costs during the study period

Country	Namibia	Nigeria	Uganda
Site	Public: AB May Cancer Care Centre, Windhoek Central Hospital	Public: Federal Medical Centre Owerri and Abia State University Teaching Hospital; Private: Marantha clinic, Aba	Public: Mulago Hospital and Uganda Cancer Institute, Kampala
Catchment population	National	Federal	National
ABC-DO participants*	104 non-black and 398 black women	313 public, 80 private patients	430
Medical treatment: approximate patient out-of-pocket costs in USD, at time of study			
Imaging			
Mammography (diagnostic)	Free	22	30
Computer tomography	Free	145	100
Biopsy^A^	Free	85	10
Fine-needle aspiration cytology (FNAC)^A^	Free	6	10
Immunohistochemistry (IHC)^AB^	Free	Not available	70
Full blood tests	Free	40	5
ECG	Free	90	20
Mastectomy	Free	250	Free
Pre-chemotherapy tests	Free	No information	50–400
Chemotherapy per cycle	Free	65–1500	120–200
Hormone drugs/month			
Tamoxifen	Free	40	10–20
Anastrazole	Free	20	50–60
Herceptin	Free	1100	3000
Radiotherapy^c^	Free	Nearest public radiotherapy facility is 150 km away from Enugu	Free^c^

^*^Numbers refer to excluded women without any treatment data (*n* = 7)

^A^Specimens were taken for all women in Namibia and 94% and 59% of Ugandan and Nigerian patients, respectively. Most specimens were core biopsies (74%), followed by excisional biopsies (14%) and Fine Needle Aspiration Cytology (FNAC) (11%). IHC was performed for 96% of Namibian women, but only 10% and 9% of Ugandan and Nigerian women, respectively

^B^Prices are for determination of full receptor status (endocrine, progesterone and human epidermal growth factor receptor 2)

^c^Radiotherapy was not available in Uganda from March 2016 for 1.5 years

### Treatment data

Due to the complexity of multimodality breast cancer treatment, for the present analysis, we analysed whether any breast cancer treatment was initiated for each woman within 12 months of diagnosis (yes/no), which we defined as having had or commenced at least one of surgery, radiotherapy or systemic (chemotherapy or endocrine) therapy. Palliative care without curative intent (e.g. opioids for pain management) was also recorded, but was not included here. The binary treatment indicator was created as follows: First, data was extracted from two systematically recorded data sources—medical records and patient self-reporting.

(i) Medical records were collected by medical personnel of the hospital, separately for surgery (*n* = 523), chemotherapy (*n* = 712), endocrine therapy (*n* = 603) and radiotherapy (*n* = 324). Medical records were supposed to be filled in after each medical consultation for one of the treatments, to capture the medical decision of both intended treatments and those actually received (multiple records per woman are possible): These were used to capture information on treatment recommendation, date of administration, completion and specific type (e.g. specific drug, number of cycles). Research nurses were prompted by the study’s tailor-made mHealth application, which was used for all aspects of data collection, to obtain such data periodically.
(ii) Self-reports: at their 6-month follow-up interview (*n* = 1051 women interviewed; 133 (10%) died beforehand), women were asked whether they had had each of breast surgery, chemotherapy, radiotherapy or other treatment and, if not, the reason therefor.

Secondly, data from these two sources was combined to create a single indicator of treatment initiated (yes/no). Thereafter, for women for whom there was no indication of treatment received from either source, all text fields of patient follow-up calls, where interviewers could enter any additional information, were searched for any indication of treatment status (*n* = 77 additional treatment records identified). Thereafter, if there was disagreement in the information obtained from the medical records and the self-reported data, the woman was considered to have initiated treatment if the most recent information indicated so: e.g. if there was no treatment indicated in the medical records at a certain time of data entry, but self-report data entered at a later point in time indicated treatment, the patient was considered treated. If the most recent information did not report any treatment, the information of the medical record was considered correct. The 77 women whose treatment information was obtained via open text field entries were considered treated.

### Determinants of treatment received

Socio-economic, breast-specific and other health-related determinants were examined in relation to treatment received. These included the following: (i) five “population groups” defined according to country (Namibia, Nigeria, Uganda), ethnicity (black vs. non-black) in Namibia and hospital type (private vs public) in Nigeria; (ii) recorded TNM stage at diagnosis: stages I/II combined (as stage I was rare), stage III, stage IV and unknown stage; (iii) age at diagnosis (< 40 years, 40–49, 50–59, 60–69 and ≥ 70); (iv) country-specific socio-economic position (SEP) categories (low, middle and high) which were constructed based on thirds of each country’s distribution of a SEP score derived from combining the following self-reported possessions and facilities: home ownership, indoor water, flush toilet, electricity, vehicle, refrigerator, landline, gas or electric stove and a bed; (v) belief in traditional medicine (yes/no); (vi) employment status as highly skilled/skilled or unskilled/not applicable (containing, e.g. the informal work sector and housewives); (vii) body mass index (BMI) derived from measured height and weight at recruitment (five categories: < 18.5, 18.5 to < 25, 25 to < 30, ≥ 30 kg/m^2^ and unknown); (viii) residential area, i.e. living in an urban (city/town) or rural (village/rural); (ix) previous breast cancer knowledge (ever/never heard of the disease prior to current disease onset); (ix) belief in traditional medicine (yes/no); (xi) belief in spiritual healing (yes/no) and (xii) self-reported HIV status (yes/no).

At the 6-month follow-up interview, we also ascertained a woman’s, and her family’s, out-of-pocket healthcare costs in the past 3 months in the two countries without free cancer care, i.e. Nigeria and Uganda. Answers were provided in the local currency and subsequently converted into US dollars.

### Statistical methods

Associations between differences in healthcare systems and treatment were captured by between-country comparisons in treatment received. Within-country determinants of treatment received were identified by fitting logistic regression models. Minimally adjusted models were first fitted to all women combined, adjusting for ABC-DO population group, breast cancer stage and age at diagnosis. In addition, a fully adjusted model further included the variables SEP, BMI and residential area. Population-group heterogeneity was assessed post hoc via fitting separate stage and age-adjusted models for Ugandan and Nigerian population groups (models not fitted for the Namibian population groups due to the negligible proportion of untreated women in these population groups). All analyses were performed in STATA version 14.2. All *P* values are two-sided at a significance level of 0.05. Sensitivity analyses of determinants of treatment initiation were conducted excluding women with stage IV breast cancer and results did not change (results not shown).

## Results

### Study population

Among the 1335 women included, mean age at breast cancer diagnosis was 50.7 years (SD = 13.6, Additional file 1: Table S1). Overall, 597 (45%) of women were diagnosed at stage III and 204 (15%) at stage IV, with a markedly improved stage distribution in Namibian non-black women. Most women had unskilled jobs (*n* = 923; 70%), and other than in Uganda, where 74% (*n* = 320) of women lived in rural areas, the majority resided in urban areas. Nine percent (*n* = 121) were HIV-positive. A higher percentage of women reported believing in conventional medicine (94%) than in spiritual healing (66%) or traditional medicine (24%).

### Percentage of women who had not initiated cancer treatment

**Fig. 1:**
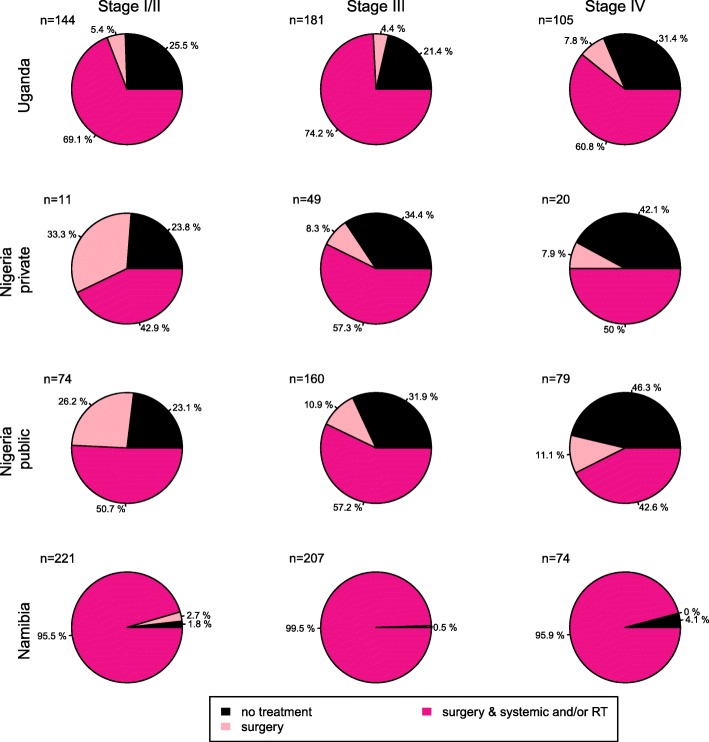
Treatment regimens provided within 1 year of breast cancer diagnosis by population group in the ABC-DO cohort study

Treatment information was known for 1325 (99%) of women, of whom 83% (*n* = 1098) had initiated breast cancer treatment within 1 year of diagnosis and 17% (*n* = 227) had not (Table 2). These percentages varied greatly between settings, with the highest percentage of untreated women in the two regional hospitals in Nigeria, at 38% and 34% in the public and private settings respectively, 17% at the national referral hospital in Uganda and was near zero in the Namibia Cancer Care Centre (0% in non-black women and 1.3% in black women; Fig. 1). Treatment was mostly in the form of surgery and/or chemotherapy, whilst treatment also included radiotherapy for two thirds of women at the Namibian Cancer Care Centre, 15% of women in Uganda—during a period when the radiotherapy machine was not in operation for 18 months—and < 5% of women at the Nigerian regional hospitals.

**Table 2:** Breast cancer treatment initiation within 1 year of diagnosis in the ABC-DO cohort: percentage untreated and odds ratios for initiating treatment associated with socio-demographic, comorbidities and stage at diagnosis

		Women not treated 227 (17.2%)	Women treated 1098 (82.8%)	Odds ratios for initiating treatment within 12 months of diagnosis		Test for interaction with population group
		*N* (row %), all sites combined		OR (95% CI)^A^	OR (95% CI)^B^	*p*
Stage at breast cancer diagnosis						0.408
I and II		47 (10.4)	403 (89.6)	1	1	
III		101 (16.9)	496 (83.1)	0.86 (0.57 to 1.28)	0.96 (0.63 to 1.45)	
IV		41 (20.1)	163 (79.9)	0.60 (0.36 to 0.98)	0.63 (0.37 to 1.07)	
Unknown		38 (51.4)	36 (48.6)	0.26 (0.14 to 0.45)	0.26 (0.14 to 0.48)	
Age at diagnosis						0.640
< 40		64 (20.9)	243 (79.1)	0.70 (0.44 to 1.10)	0.68 (0.42 to 1.10)	
40–< 50		65 (17.4)	308 (82.6)	0.83 (0.53 to 1.30)	0.77 (0.48 to 1.23)	
50–< 60		46 (14.3)	276 (85.7)	1	1	
60–< 70		32 (16.2)	165 (83.8)	0.76 (0.44 to 1.30)	0.74 (0.42 to 1.31)	
≥ 70		20 (15.8)	107 (84.2)	0.48 (0.25 to 0.93)	0.57 (0.29 to 1.16)	
SEP						0.917
Low		129 (21.5)	408 (78.5)	1	1	
Middle		56 (15.1)	382 (84.9)	1.20 (0.81 to 1.79)	1.17 (0.77 to 1.76)	
High		42 (11.8)	246 (88.2)	2.40 (1.56 to 3.69)	2.45 (1.53 to 3.90)	
Employment						0.941
Unskilled		172 (18.8)	751 (81.2)	0.70 (0.48 to 1.02)	0.83 (0.56 to 1.23)	
Skilled		55 (13.8)	348 (86.2)	1	1	
BMI	(kg/m^2^)					0.023
< 18.5		9 (11.6)	124 (88.4)	1.58 (0.70 to 3.59)	1.83 (0.79 to 4.21)	
18.5–25		103 (20.4)	404 (89.6)	1	1	
25–30		67 (17.5)	315 (82.5)	1.09 (0.74 to 1.59)	0.96 (0.65 to 1.41)	
30+		35 (11.5)	269 (88.5)	1.76 (1.10 to 2.81)	1.53 (0.95 to 2.47)	
Residential area						0.123
Urban		112 (16.5)	569 (83.5)	1	1	
Rural		115 (17.9)	530 (82.1)	0.88 (0.61 to 1.25)	1.07 (0.73 to 1.56)	
Breast cancer knowledge						0.286
Yes		170 (15.8)	905 (84.2)	1	1	
No		57 (22.7)	194 (77.3)	0.80 (0.54 to 1.17)	0.81 (0.55 to 1.21)	
Belief in traditional medicine						0.013
Yes		80 (25.2)	238 (74.8)	0.63 (0.44 to 0.89)	0.67 (0.47 to 0.97)	
No		147 (14.6)	860 (83.4)			
Belief in spiritual healing						0.004
Yes		149 (17.4)	727 (82.6)	1.18 (0.83 to 1.68)	1.21 (0.84 to 1.21)	
No		78 (17.0)	371 (83.0)	1		
HIV status^C^						n.a.
Negative		61 (16.1)	1143 (83.9)	1	1	
Positive		16 (32.0)	39 (68.0)	0.39 (0.19 to 0.79)	0.33 (0.16 to 0.70)	

*BC* breast cancer, *BMI* body mass index, *CI* confidence interval, *OR* odds ratio, *SEP* socio-economic position

^A^OR adjusted for the ABC-DO population group, breast cancer stage at diagnosis and age at diagnosis

^B^OR adjusted for the ABC-DO population group, breast cancer stage at diagnosis, age at diagnosis, SEP, BMI and residential area

^C^Logistic regression models restricted to the Ugandan women (*N* = 430)

**Fig. 2:**
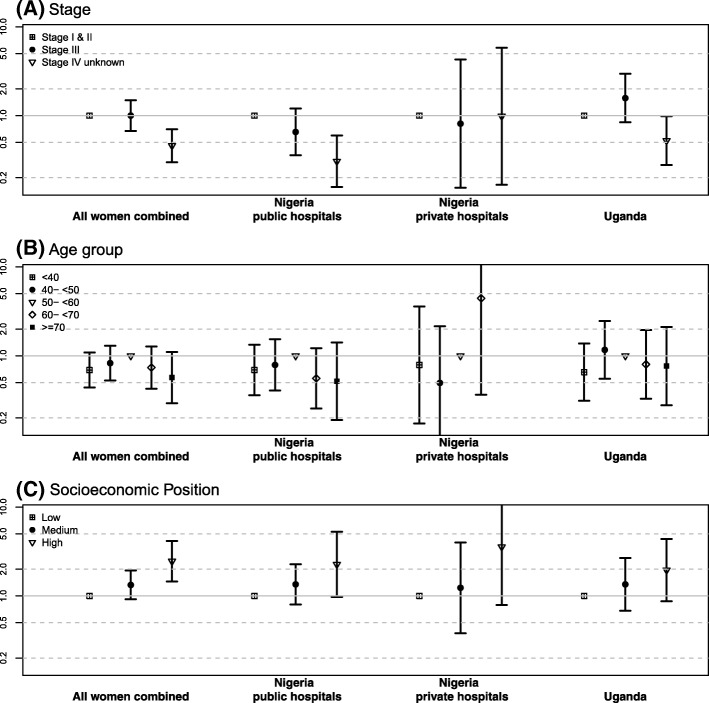
Odds ratios and 95% confidence intervals for receiving treatment by **a** breast cancer stage, **b** age at diagnosis and **c** socio-economic position, for all women combined and separately for Uganda, and for public and private hospitals in Nigeria. **a** Adjusted for age at diagnosis, **b** adjusted for breast cancer stage and **c** adjusted for age and stage at diagnosis. Models for all women combined additionally adjusted for population group

Odds ratios of associations with receipt of cancer treatment are provided in Table 2, and those for age, stage and SEP are plotted in Fig. 2.

### Stage

In Nigeria, percentages of untreated women increased linearly with more advanced cancer stage, with 23% (absolute) more untreated women in stage IV vs stage I/II, whereas in Uganda only stage IV (and not stage III) had a higher (by 3%) percentage of untreated women. For stage III cancer, this association was fully attenuated after adjusting for SEP, BMI and residential area, thus in the fully adjusted model, as expected, only stage IV women had lower treatment odds (stage IV v stage I/II: 0.63, 95% CI 0.37, 1.07, Table 2). In both settings, among the 8% women with missing stage information, untreated percentages were particularly high, at 51%, which was not explained by adjustments made (OR 0.26; 0.14, 0.45).

### Age

An inverse U-shaped association between age and treatment was observed in both settings, with percentages receiving treatment being highest among 50–59-year-old women. Women aged 70 and over, comprising 6% of patients, were least likely to be treated, with an absolute difference of 15% in the Nigerian public setting and 10% in Uganda (OR 0.48, 95% CI 0.25, 0.93) (Additional file 3: Figure S1, Additional file 2: Table S2). For young women (< 40 years), who represented 26% of patients, treated percentages were 4% less in Uganda and 8% less in Nigeria (stage and age-adjusted OR 0.70; 95% CI 0.44, 1.10). Further adjustment for BMI, SEP and area of residence partially attenuated the associations, but the slight inverse U-shape remained. These associations held when restricted to HIV-negative women and were not restricted to women with low SEP or later-stage disease; instead, they were slightly stronger in early-stage disease and in medium and high SEP groups (data not shown).

### Socio-economic factors

**Fig. 3:**
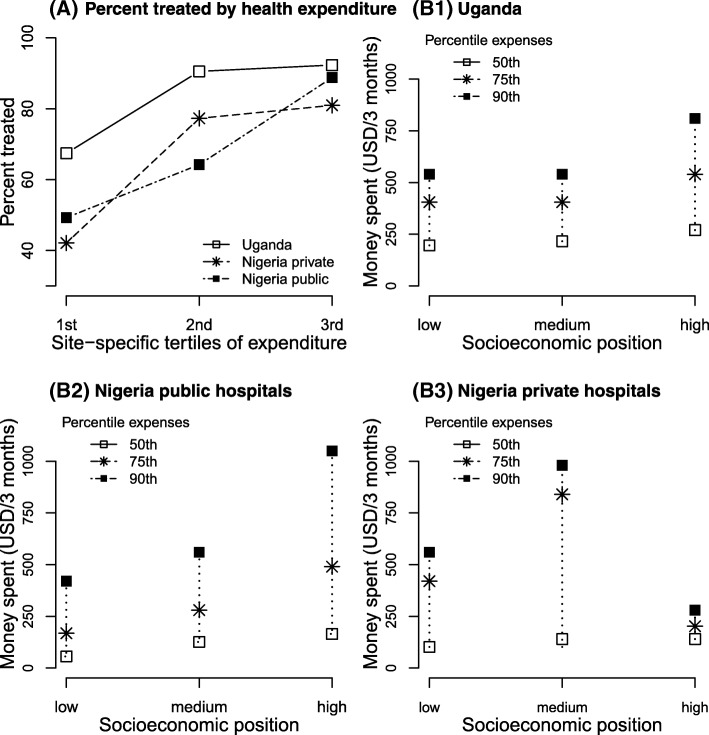
Household health-related expenses associated with breast cancer treatment. **a** The percentage of treated women per setting-specific tertile of household breast cancer expenditure. Tertiles of expenditure relate to the site-specific 3-monthly health-related household expenses and may be interpreted as low, medium and high levels of expenditure. **b1**–**b3** Household health-related expenses during the last 3 months corresponding to 50th, 75th and 90th percentile cut-offs for women in low, medium and high socio-economic positions, by an ABC-DO setting. Local currencies were converted to USD. The gross national income (GNI) per capita in 2017 was 600 USD in Uganda and 2100 USD in Nigeria

A consistent and marked socio-economic gradient in the percentage of untreated women was seen (Fig. 3). Absolute percentages of untreated women in the lowest vs highest SEP tertiles were 21% vs. 11% in the Uganda national referral hospital, 41% vs. 21% and 43% vs. 28% in the two Nigerian regional settings (Additional file 3: Figure S1, Additional file 2: Table S2). This SEP-treatment association was unaltered after further adjustment for BMI and residential area (fully adjusted OR 2.45; 95% CI 1.53, 3.90; Table 2). The association with skilled/unskilled employment was in a similar direction, albeit not statistically significant, whilst there was no clear association between residential area and treatment.

### Comorbidities

Obese (BMI > 30 kg/m^2^) and, to a lesser extent and non-significant, underweight (BMI < 18.5 kg/m^2^) women each had a higher odds of receiving breast cancer treatment compared to those with a normal BMI (18.5–25 kg/m^2^) (age and stage-adjusted OR 1.76; 95% CI 1.10, 2.81 and 1.58; 0.70, 3.59) respectively; Table 1). Further adjustment for SEP and residential area slightly weakened the obesity association (fully adjusted OR 1.53; 95% CI 0.95, 2.47) but strengthened the underweight association (1.83; 0.79, 4.21). Regarding HIV, after full adjustment, HIV-positive women had one third of the odds of being treated relative to their HIV-negative counterparts (Table 2), reflecting the Ugandan situation, yet almost all Namibian black women, the group with the highest (14%) HIV prevalence (Additional file 1: Table S1), received breast cancer treatment. In Uganda, the total percentage difference between positive and negative women was 16%.

### Health knowledge and beliefs

Health beliefs affected treatment received, but the effects were setting-specific (*p* values for the interaction in Table 2). In Uganda, where 38% of women reported believing in traditional medicine, such women were less likely to receive treatment (27% untreated vs 13% untreated among non-believers (Additional file 2: Table S2); fully adjusted OR 0.39; 95% CI 0.22, 0.66 (population group-specific ORs not shown)). Further, in the 6-month follow-up interview, 40% of these women stated that they were currently attending a traditional healer for treatment. These were eight times more likely not to have received conventional breast cancer treatment (7.84; 3.64, 16.91). In contrast, in the Nigerian private hospital, the odds of being treated for breast cancer was higher among women who believed in traditional medicine (fully adjusted OR 5.1; 95% CI 1.1, 24.7) or in spiritual healing (6.5; 1.2, 34.5) than among their non-believer counterparts, with the wide CIs reflecting the small sample size. Finally, previous breast cancer knowledge was not associated with treatment overall, but had an effect in Uganda, where untreated percentages were lower in women who knew that breast cancer was curable than those who did not (13% v 21%).

### Self-reported treatment costs and reasons for lack of treatment

Self-reported information on treatment gaps was available for 131 women who did not undergo surgery despite medical recommendation; the reasons for refusing surgery were cost (47%, *n* = 62) and personal decision (e.g. due to lack of belief in its effectiveness, fear or non-compliance to or rejection of therapy) (31%, *n* = 40). In addition, for 55 women, a medical reason for not getting surgery was recorded in the clinical notes (e.g. too old and frail, tumour too advanced).

Healthcare expenses were paid out-of-pocket by the patient herself (ranging from 42% in Uganda to 67% in Nigerian public hospitals) or by family and friends (65%, all settings). Median household healthcare expenses in a 3-month snapshot period increased with increasing SEP (Fig. 3B1-B3: range $196 to 270 in Uganda, $56 to 224 in Nigerian public and $102 to 140 in Nigerian Private hospitals) and tended to be associated with increasing percentages of treated women, particularly so for Nigerian public hospitals (Fig. 3B2). Although only asked for a 3-month interval, it is likely that the cumulative costs will exceed the annual gross national income per capita of 600 USD in Uganda and 2100 USD in Nigeria (2017 census; https://data.worldbank.org). Untreated women also incurred healthcare expenses, with median household expenses of 83, 20 and 55 USD, respectively, for women in Uganda, Nigeria public and Nigeria private hospitals (all SEPs combined).

## Discussion

In this prospective African breast cancer cohort, we found large between-setting differences in cancer treatment access. Whilst almost all women who attended a specialised Cancer Care Centre in Namibia received treatment regardless of race and SEP, one in six patients at Uganda’s major referral hospital and one in three patients at two regional settings in Nigeria had not initiated any type of cancer treatment within 1 year of diagnosis. In Uganda and Nigeria, countries without universal health care coverage, financial barriers, ageing, HIV positivity and traditional medicine health beliefs and a slight suggestion that young age (< 40) increased treatment gaps. The absence of a clear difference in treatment rates between stage IV compared to stage I–III patients among all settings highlights the need for cancer care education in the SSA region (percentages of untreated women per stage displayed in Additional file 2: Table S2).

### Plausibility and comparison with other studies

The treatment divide between ABC-DO countries reflects, to a large extent, the marked differences in healthcare systems and out-of-pocket costs to women and their families. In Namibia, where cancer treatment is free, almost all women attending the cancer care hospital were willing to be treated. In contrast, in Uganda and Nigeria, cancer care costs are paid out-of-pocket by the patients, often with the help of relatives and friends. Accordingly, SEP was the most influential determinant of treatment access, in line with, but of larger magnitude than, other studies linking social status to cancer outcomes [14–17]. Expenditure levels amounted to financial catastrophe, which have also been reported for other LMIC, mainly in the Asian region [8, 9].

We found slight inverse U-shaped relationship of treatment with age, which, to the best of our knowledge, has not been reported so far and warrants investigation in other settings. Elderly women may not receive treatment due to their frail physical condition or patients’ preference [18, 19]. The lower treatment percentage seen in younger women is of concern and possibly arises in these settings due to stigma, fear of disfigurement and abandonment. Preference for traditional medicine also prevented women from initiating cancer treatment in Uganda. Feelings of stigmatisation, hopelessness and fatalism are commonly reported by patients diagnosed with breast cancer in SSA [20, 21]. Such feelings, together with lack of knowledge of the disease, contribute to rejection of allopathic cancer treatments and preference for traditional medical practitioners (TMPs), who are often cancer illiterate and do not take the adequate action [22].

Increases in life expectancy of HIV-positive women combined with rises in the incidence of breast cancer leads to an increasing number of women in SSA with the double HIV-breast cancer burden. In Uganda, HIV-positive women were less likely to get treatment than their HIV-negative counterparts, but the reasons for this were unknown. A personalised treatment management plan is needed to minimise possible drug-drug interactions of chemotherapy with ARVs in the HIV-positive breast cancer patient [23]. Non-medical reasons for the lower treatment proportions rates might include stigmatisation as well as poor treatment literacy of the patients and health care workers.

### Study strengths and limitations

The study benefits from detailed epidemiologic and time-indexed treatment data, collected via a mHealth application which enabled prospective collection of standardised data across multiple sites, including prompts to interviewers for data extraction and follow-up interviews. The reliability of data on whether treatment was received was strengthened by the multiple sources—in particular that of the 1098 (83%) women that have been considered as treated across the sites, this information was confirmed by the women for 66% (*n* = 880), thus reducing the possibility for incorrect overestimation due to mislaid case notes to 17%. Nevertheless, the latter factor may have affected the seemingly higher percentage untreated in women with unknown stage.

A limitation of the present study is the difficulty to disentangle whether untreated proportions are rather attributable to a country or the specific hospital setting in this specific country. This is due to the hospital-based nature of ABC-DO, and hence, the treatment proportions reported reflect those among women who reach this level of the cancer care system in a given country. This is particularly relevant for the Namibian specialised Cancer Care Centre, where the high treated percentages are among women who have already made the journey to this national referral centre. Nevertheless, the high treated proportions in this centre also reflect willingness of women to receive treatment when the burden of treatment costs is alleviated from the patient. Further, the national hospital referral network in Namibia might heighten the chance of a patient to reach cancer care at a specialised centre. In every setting, breast cancer patients who do not reach this level of the health system and may be more likely to go untreated, are not included. Such patients may be substantial in number as various patient-level and health system-level barriers prolong the journey to breast cancer diagnosis [12], thus overall untreated proportions may be even higher. We do, however, expect that the profile of women vulnerable to under/no treatment are captured in the associations observed.

### Health system implications

The study identified marked inequities in access to breast cancer treatment in SSA settings. The much lower treatment rates in Uganda and Nigeria, when compared to Namibia, underline the importance of providing population-wide affordable cancer care through universal healthcare coverage (UHC) and a national hospital referral network. Even in the absence of UHC, knowledge that lower social groups are vulnerable to treatment gaps should be addressed, and navigational, financial and educational support provided to reduce these. Appropriate treatment facilities across the continuum of cancer diagnosis and care are currently lacking in most SSA countries [15, 24, 25]. In a mathematical cost-effectiveness modelling of cancer treatment in SSA, BC treatment of all stages has been shown cost-effective if also accompanied by early detection programs [26]. In particular at earlier stages, treatment is more cost-effective, as treatment is less expensive with higher chances to cure. Costs of BC treatment in Africa were estimated at $78 per disability-adjusted life year (DALY) for treating early-stage breast cancer with surgery and radiotherapy vs. $4986 per DALY for treating metastatic BC with systemic chemotherapy. In this context [27], the high percentage of patients who were not treated within the first year of diagnosis though diagnosed at earlier stages, especially stage II, needs to be urgently addressed.

The present results also highlight the need to consider how to integrate the informal health care sector into cancer care, particularly in countries where traditional medical practitioners (TMPs) serve a large part of the population. TMPs are a significant source of health care delivery in cancer management in SSA [22]. On average, one TMP serves 400 persons, contrasting with one allopathic practitioner per 12,000 persons and two surgeons per 100,000 persons [28]. In a survey asking TMPs about cancer management, their literacy was still limited: the majority of TMPs recognised cancer, but only at advanced stages [22]. Further, most TMPs believed that they could treat cancer; thus, appropriate referral for the specialised diagnosis and care needed for breast cancer would be delayed. Thus providing training courses on breast cancer awareness and recognition, and the importance of immediate hospital referral and treatment initiation and completion, is a potential promising approach to significantly improve access to breast cancer diagnosis and treatment in SSA. Such educational programs need to include the affected breast cancer patients themselves, but also TMPs and conventional health care providers at the frontline and at key referral nodes to both accelerate the route to diagnosis and increased treatment access.

## Conclusion

In conclusion, the present results provide up-to-date empirical data on woman’s access to breast cancer treatment in SSA, and on the key role played by major financial and some sociocultural barriers. The marked between-country divide in treatment access, coupled with the marked and consistent within-population socio-economic differentials in the proportion of untreated patients, supports the call for universal free access to cancer diagnosis and treatment in SSA to prevent growing social inequities in breast cancer care and survival in the region.

## Additional files

Additional file 1: **Table S1.** provides a descriptive table of the ABC-DO cohort, all women combined and by study population group, and the predictors used in the present analysis. (DOCX 28 kb)

Additional file 2: **Table S2.** provides a descriptive table of the predictors used in the present analysis restricted to the untreated women. (DOCX 28 kb)

Additional file 3: **Figure S1.** shows the proportion of breast cancer patients whose cancer treatment had not been initiated within 12 months of diagnosis, by selected socio-demographic factors and settings. (PDF 392 kb)

## Data Availability

Data are available from ABC-DO PIs, email: abc-do@iarc.fr

## Abbreviations

ABC-DO: African Breast Cancer - Disparities in Outcomes cohort study
BMI: Body mass index
CI: Confidence interval
HER2: Human epidermal growth factor receptor 2
HIV: Human immunodeficiency virus
IHC: Immunohistochemistry
OR: Odds ratio
SD: Standard deviation
SEP: Socio-economic position
SSA: Sub-Saharan Africa
TMP: Traditional medical practitioners
TNM: Tumour, node, metastasis classification
