# Supplementary material for: Inequities in breast cancer treatment in sub-Saharan Africa: findings from a prospective multi-country observational study
Source: Breast Cancer Res. 2019 Aug 13;21:93. doi: 10.1186/s13058-019-1174-4 (PMC6691541; doi:10.1186/s13058-019-1174-4)
Supplement: Supplementary file 1 — Table S1. provides a descriptive table of the ABC-DO cohort, all women combined and by study population group, and the predictors used in the present analysis. (DOCX 28 kb) [file 13058_2019_1174_MOESM1_ESM.docx]

| **Additional file 1: Table S1:** Counts and percentages for BC treatment and different sociodemographic characteristics amongst the study sites | | | | | | | |
| --- | --- | --- | --- | --- | --- | --- | --- |
|  |  | **All** | **Namibia black** | **Namibia non-black** | **Nigeria public** | **Nigeria private** | **Uganda** |
|  |  | **n (%)** | **n (%)** | **n (%)** | **n (%)** | **n (%)** | **n (%)** |
| **N total** | | **1325 (100)** | **398 (100)** | **104 (100)** | **313 (100)** | **80 (100)** | **430 (100)** |
|  | Treated | 1098 (82.8) | 393 (98.7) | 104 (100) | 195 (62.1) | 53 (66.3) | 353 (82.1) |
| **Stage** | |  |  |  |  |  |  |
|  | I & II | 450 (34.0) | 143 (35.9) | 78 (75.0) | 74 (23.6) | 11 (13.8) | 144 (33.5) |
|  | III | 597 (45.1) | 188 (47.2) | 19 (18.3) | 160 (51.1) | 49 (61.3) | 181 (42.1) |
|  | IV | 204 (15.4) | 67 (16.8) | 7 (6.7) | 45 (14.4) | 15 (18.8) | 70 (16.3) |
|  | unknown | 74 (5.6) | 0 (0) | 0 (0) | 34 (10.9) | 5 (6.3) | 35 (8.1) |
| **Age** |  |  |  |  |  |  |  |
|  | < 40 | 307 (23.2) | 81 (20.4) | 7 (6.7) | 79 (25.2) | 24 (30.0) | 116 (27.0) |
|  | 40- <50 | 373 (28.1) | 103 (25.9) | 23 (22.1) | 83 (26.4) | 31 (38.8) | 133 (30.9) |
|  | 50- <60 | 322 (24.3) | 100 (25.1) | 28 (26.9) | 83 (26.4) | 16 (20.0) | 95 (22.1) |
|  | 60- <70 | 197 (14.9) | 60 (15.1) | 27 (26.0) | 47 (15.0) | 9 (11.3) | 54 (12.6) |
|  | >= 70 | 127 (9.6) | 54 (13.6) | 19 (18.3) | 22 (7.0) | 0 (0) | 32 (7.4) |
| **SEP** ^A^ |  |  |  |  |  |  |  |
|  | low | 600 (45.3) | 177 (44.5) | 0 (0) | 140 (44.6) | 29 (36.3) | 254 (59.1) |
|  | middle | 438 (33.0) | 136 (34.2) | 42 (40.4) | 137 (43.6) | 32 (40.0) | 91 (21.2) |
|  | high | 288 (21.7) | 85 (21.4) | 62 (59.6) | 37 (11.8) | 19 (23.8) | 85 (19.8) |
| **Employment** | |  |  |  |  |  |  |
|  | unskilled / n.a.^B^ | 923 (69.6) | 298 (74.9) | 33 (31.7) | 216 (68.8) | 48 (60.0) | 328 (76.3) |
|  | skilled | 403 (30.4) | 100 (25.1) | 71 (68.3) | 98 (31.2) | 32 (40.0) | 102 (23.7) |
| **BMI** ^C^ |  |  |  |  |  |  |  |
|  | <18.5 | 133 (10.0) | 61 (15.3) | 8 (7.7) | 37 (11.8) | 3 (3.8) | 24 (5.6) |
|  | 18.5-<25 | 507 (38.2) | 129 (32.4) | 32 (30.8) | 125 (39.8) | 28 (35.0) | 193 (44.9) |
|  | 25-<30 | 382 (28.8) | 104 (26.1) | 21 (20.2) | 87 (27.7) | 19 (23.8) | 151 (35.1) |
|  | 30+ | 304 (22.9) | 104 (26.1) | 43 (41.4) | 65 (20.7) | 30 (37.5) | 62 (14.4) |
| **Residential area** | |  |  |  |  |  |  |
|  | urban | 681 (51.4) | 224 (56.3) | 93 (89.4) | 186 (59.2) | 68 (85.0) | 110 (25.6) |
|  | rural | 645 (48.6) | 174 (43.7) | 11 (10.6) | 128 (40.8) | 12 (15.0) | 320 (74.4) |
| **BC knowledge** ^D^ | |  |  |  |  |  |  |
|  | yes | 1075 (81.1) | 335 (84.2) | 103 (99.0) | 231 (73.6) | 69 (86.3) | 337 (78.4) |
|  | no | 251 (18.9) | 63 (15.8) | 1 (1.0) | 83 (26.4) | 11 (13.8) | 93 (21.6) |
| **Belief in traditional medicine** | | |  |  |  |  |  |
|  | yes | 318 (24.0) | 56 (14.1) | 7 (6.7) | 68 (21.7) | 22 (27.5) | 165 (38.4) |
|  | no | 1007 (76.0) | 342 (85.9) | 97 (93.3) | 245 (78.3) | 58 (72.5) | 265 (61.6) |
| **Belief in spiritual healing** | |  |  |  |  |  |  |
|  | yes | 876 (66.1) | 297 (74.6) | 70 (67.3) | 233 (74.4) | 69 (86.3) | 207 (48.1) |
|  | no | 449 (33.9) | 101 (25.4) | 34 (32.7) | 80 (25.6) | 11 (13.8) | 223 (51.9) |
| **HIV status** | |  |  |  |  |  |  |
|  | negative | 1204 (90.9) | 343 (86.2) | 101 (97.1) | 306 (97.8) | 74 (92.5) | 380 (88.4) |
|  | positive | 121 (9.1) | 55 (13.8) | 3 (2.9) | 7 (2.2) | 6 (7.5) | 50 (11.6) |
| \| BC: breast cancer; BMI: body mass index; SEP: socio-economic position \| \| \| --- \| --- \| \| ^A^ Country-specific socioeconomic position (SEP) categories (low, middle and high) which were constructed based on thirds of each country’s distribution of a SEP score derived from combining the following self-reported possessions and facilities: home ownership, indoor water, flush toilet, electricity, vehicle, refrigerator, landline, gas or electric stove and a bed  ^B^ 160 women in the category "Not applicable" comprising e.g. housewifes and the informal work sector, were considered unskilled.  ^C^ 53missing values of BMI within the population  ^D^ Breast cancer (BC) knowledge retrieved via the question “Have you ever heard of BC? (yes/no)” \| | | | | | | | |
