# Supplementary material for: Inequities in breast cancer treatment in sub-Saharan Africa: findings from a prospective multi-country observational study
Source: Breast Cancer Res. 2019 Aug 13;21:93. doi: 10.1186/s13058-019-1174-4 (PMC6691541; doi:10.1186/s13058-019-1174-4)
Supplement: Supplementary file 2 — Table S2. provides a descriptive table of the predictors used in the present analysis restricted to the untreated women. (DOCX 28 kb) [file 13058_2019_1174_MOESM2_ESM.docx]

| **Additional file 2: Table S2:** ABC-DO overall and site-specific frequencies and percentages of breast cancer patients whose cancer treatment had not been initiated within 1 year of diagnosis: overall and stratified by sociodemographic and cancer characteristics. Percentages relate to the site and category-specific percentage of untreated women, thus allowing for immediate comparisons between categories for a given site. | | | | | | |
| --- | --- | --- | --- | --- | --- | --- |
|  |  | **Total not treated^A^** | **Namibia black (n=398)** | **Nigeria public (n=314)** | **Nigeria private (n=80)** | **Uganda (n=430)** |
|  |  | n(row %) | n (row %) | n (row %) | n (row %) | n (row %) |
| **Not initiated treatment** | | **227 (17.2 %)** | **5 (1.3 %)** | **118 (37.7 %)** | **27 (33.8 %)** | **77 (17.9 %)** |
| **Stage** | |  |  |  |  |  |
|  | I & II | 47 (12.6) | 2 (1.4) | 17 (23.0) | 4 (36.4) | 24 (16.7) |
|  | III | 101 (17.5) | 1 (0.5) | 59 (36.9) | 17 (34.7) | 24 (13.3) |
|  | IV | 41 (20.8) | 2 (3.0) | 21 (46.7) | 4 (26.7) | 14 (20.0) |
|  | unknown | 38 (51.4) | n.a. | 21 (61.8) | 2 (40.0) | 15 (42.9) |
| **Age** |  |  |  |  |  |  |
|  | < 40 | 64 (21.4) | 1 (1.2) | 31 (39.7) | 9 (37.5) | 23 (19.8) |
|  | 40- <50 | 65 (18.6) | 1 (1.0) | 31 (37.3) | 13 (41.9) | 20 (15.0) |
|  | 50- <60 | 46 (15.6) | 1 (1.0) | 26 (31.3) | 4 (25.0) | 15 (15.8) |
|  | 60- <70 | 32 (18.8) | 0 (0.0) | 20 (42.6) | 1 (11.1) | 11 (20.4) |
|  | ≥70 | 20 (18.5) | 2 (3.7) | 10 (45.5) | n.a. | 8 (25.0) |
| **SEP** |  |  |  |  |  |  |
|  | low | 129 (21.5) | 3 (1.7) | 60 (42.9) | 12 (41.4) | 54 (21.3) |
|  | middle | 56 (17.1) | 2 (1.5) | 29 (42.0) | 11 (34.4) | 14 (15.4) |
|  | high | 42 (14.3) | 0 (0.0) | 29 (27.9) | 4 (21.1) | 9 (10.6) |
| **Employment** | |  |  |  |  |  |
|  | Unskilled / n.a **^B^**. | 172 (19.5) | 4 (1.4) | 88 (40.9) | 18 (37.5) | 62 (18.9) |
|  | skilled | 55 (16.7) | 1 (1.0) | 30 (30.6) | 9 (28.1) | 15 (14.7) |
| **BMI^C^** | kg/m^2^ |  |  |  |  |  |
|  | <18.5 | 9 (11.7) | 0 (0.0) | 7 (35.0) | n.a. | 2 (13.3) |
|  | 18.5-<25 | 103 (21.7) | 4 (3.1) | 53 (42.7) | 6 (21.4) | 40 (20.7) |
|  | 25-<30 | 67 (18.6) | 0 (0.0) | 29 (33.3) | 10 (52.6) | 28 (18.5) |
|  | 30+ | 35 (13.4) | 0 (0.0) | 20 (30.8) | 10 (33.3) | 5 (8.1) |
| **Residential area** | |  |  |  |  |  |
|  | urban | 112 (19.0) | 1 (0.4) | 72 (38.7) | 25 (36.8) | 14 (12.7) |
|  | rural | 115 (18.2) | 4 (2.3) | 46 (36.2) | 2 (16.7) | 63 (19.7) |
| **BC knowledge** | |  |  |  |  |  |
|  | yes | 204 (18.5) | 3 (0.9) | 116 (38.0) | 24 (32.4) | 61 (16.1) |
|  | no | 23 (19.5) | 2 (3.6) | 2 (28.6) | 3 (50.0) | 16 (32.0) |
| **Belief in traditional medicine** | | |  |  |  |  |
|  | yes | 170 (17.5) | 3 (0.9) | 81 (35.2) | 25 (36.2) | 61 (18.1) |
|  | no | 57 (22.8) | 2 (3.2) | 37 (44.6) | 2 (18.2) | 16 (17.2) |
| **Belief in spiritual healing** | | |  |  |  |  |
|  | yes | 147 (16.2) | 4 (1.2) | 87 (35.5) | 23 (39.7) | 33 (12.5) |
|  | no | 80 (25.7) | 1 (1.8) | 31 (45.6) | 4 (18.2) | 44 (26.7) |
| **HIV status** | |  |  |  |  |  |
|  | negative | 78 (18.8) | 0 (0.0) | 32 (40.0) | 7 (63.6) | 39 (17.5) |
|  | positive | 149 (18.5) | 5 (1.7) | 86 (36.9) | 20 (29.0) | 38 (18.4) |
| BC: breast cancer; BMI: body mass index; SEP: socio-economic position  ^A^ Refers to the total study population of the present analysis including non-black women in Namibia. All non-black women in Namibia were treated within one year after diagnosis and are therefore not shown separately in the table  ^C^ 160 women in the category "Not applicable" comprising e.g. housewifes and the informal work sector, were considered unskilled.  ^D^ 53 missing values of BMI within the population | | | | | | |
