# Supplementary material for: Inequities in breast cancer treatment in sub-Saharan Africa: findings from a prospective multi-country observational study
Source: Breast Cancer Res. 2019 Aug 13;21:93. doi: 10.1186/s13058-019-1174-4 (PMC6691541; doi:10.1186/s13058-019-1174-4)

Additional Figure 1: Proportion of breast cancer patients whose cancer treatment had not been initiated within 12 months of diagnosis, by setting

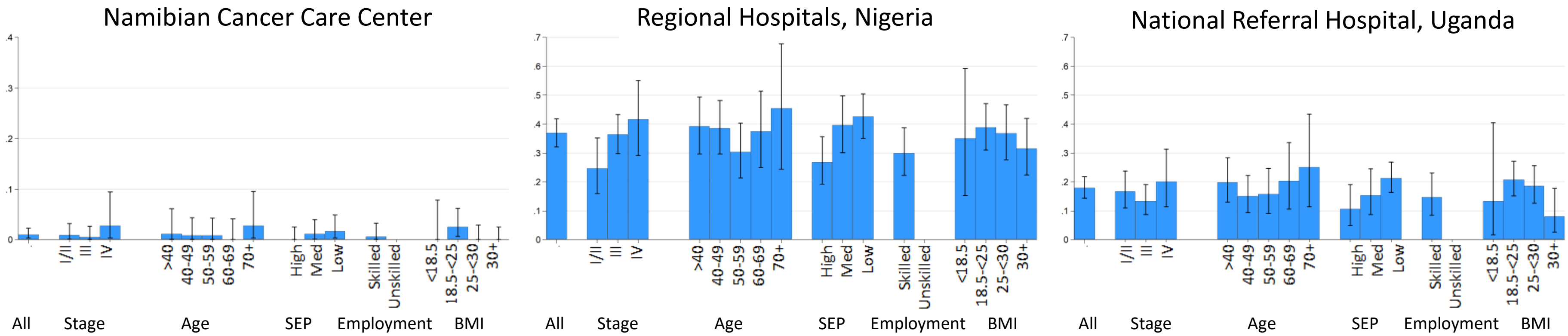

Supplement: Supplementary file 3 — Figure S1. shows the proportion of breast cancer patients whose cancer treatment had not been initiated within 12 months of diagnosis, by selected socio-demographic factors and settings. (PDF 392 kb) [file 13058_2019_1174_MOESM3_ESM.pdf]
